# 2-Amino-7,7-dimethyl-5-oxo-4-[3-(trifluoro­meth­yl)phen­yl]-1,4,5,6,7,8-hexa­hydro­quinoline-3-carbonitrile

**DOI:** 10.1107/S1600536812050684

**Published:** 2012-12-19

**Authors:** Rajni Kant, Vivek K. Gupta, Kamini Kapoor, D. R. Patil, A. G. Mulik, Madhukar B. Deshmukh

**Affiliations:** a X-ray Crystallography Laboratory, Post-Graduate Department of Physics & Electronics, University of Jammu, Jammu Tawi 180 006, India; b Department of Chemistry, Shivaji University, Kolhapur 416 004 (MS), India

## Abstract

In the title mol­ecule, C_19_H_18_F_3_N_3_O, the dihydro­pyridine and cyclo­hexene rings both adopt sofa conformations. The five essentially planar atoms of the dihydro­pyridine ring [maximum deviation = 0.039 (2) Å] form a dihedral angle of 88.19 (8)° with the benzene ring. The F atoms of the trifluoro­methyl group were refined as disordered over two sets of sites in a 0.840 (3):0.160 (3) ratio. In the crystal, N—H⋯O and N—H⋯N hydrogen bonds link mol­ecules into a two-dimensional network parallel to (100).

## Related literature

For applications of dihydro­pyridines, see: Mayler *et al.* (1989 ▶); Triggle *et al.*(1989 ▶); Leon *et al.* (2008 ▶). For related structures, see: Jiang *et al.* (2006 ▶); Tu *et al.* (2005 ▶). For ring conformations, see: Duax & Norton (1975 ▶).
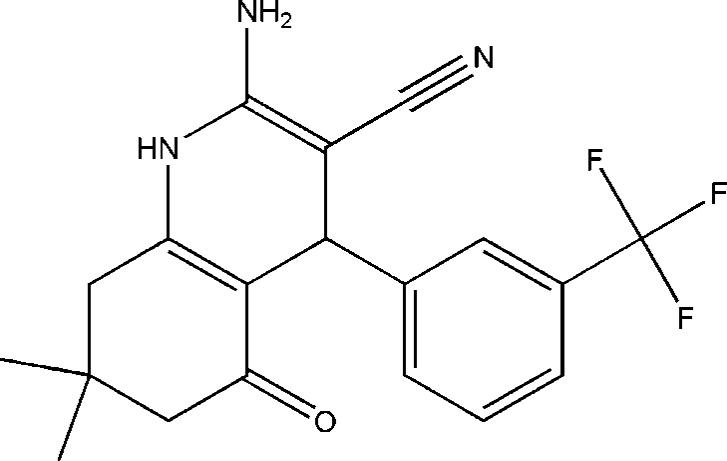


## Experimental

### 

#### Crystal data

C_19_H_18_F_3_N_3_O
*M* *_r_* = 361.36
Monoclinic, 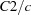

*a* = 24.2434 (6) Å
*b* = 9.6030 (2) Å
*c* = 15.2426 (4) Å
β = 93.960 (2)°
*V* = 3540.15 (15) Å^3^
*Z* = 8
Mo *K*α radiation
μ = 0.11 mm^−1^
*T* = 293 K
0.3 × 0.2 × 0.2 mm

#### Data collection

Oxford Diffraction Xcalibur Sapphire3 diffractometer
Absorption correction: multi-scan (*CrysAlis PRO*; Oxford Diffraction, 2010 ▶) *T*_min_ = 0.896, *T*_max_ = 1.000
42194 measured reflections
3469 independent reflections
2462 reflections with *I* > 2σ(*I*)
*R*_int_ = 0.059

#### Refinement

*R*[*F* ^2^ > 2σ(*F* ^2^)] = 0.057
*wR*(*F* ^2^) = 0.143
*S* = 1.03
3469 reflections
247 parameters
6 restraints
H-atom parameters constrained
Δρ_max_ = 0.31 e Å^−3^
Δρ_min_ = −0.39 e Å^−3^

### 

Data collection: *CrysAlis PRO* (Oxford Diffraction, 2010 ▶); cell refinement: *CrysAlis PRO*; data reduction: *CrysAlis PRO*; program(s) used to solve structure: *SHELXS97* (Sheldrick, 2008 ▶); program(s) used to refine structure: *SHELXL97* (Sheldrick, 2008 ▶); molecular graphics: *ORTEP-3* (Farrugia, 2012 ▶) and *PLATON* (Spek, 2009 ▶); software used to prepare material for publication: *PLATON*.

## Supplementary Material

Crystal structure: contains datablock(s) I, global. DOI: 10.1107/S1600536812050684/lh5570sup1.cif

Structure factors: contains datablock(s) I. DOI: 10.1107/S1600536812050684/lh5570Isup2.hkl

Supplementary material file. DOI: 10.1107/S1600536812050684/lh5570Isup3.cml

Additional supplementary materials:  crystallographic information; 3D view; checkCIF report

## Figures and Tables

**Table 1:** Hydrogen-bond geometry (Å, °)

*D*—H⋯*A*	*D*—H	H⋯*A*	*D*⋯*A*	*D*—H⋯*A*
N1—H1⋯O1^i^	0.86	2.39	3.117 (2)	143
N16—H16*A*⋯N20^ii^	0.86	2.12	2.966 (3)	168
N16—H16*B*⋯O1^i^	0.86	2.08	2.897 (2)	158

Symmetry codes: (i) 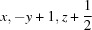; (ii) 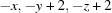.

**Fig. 1.**
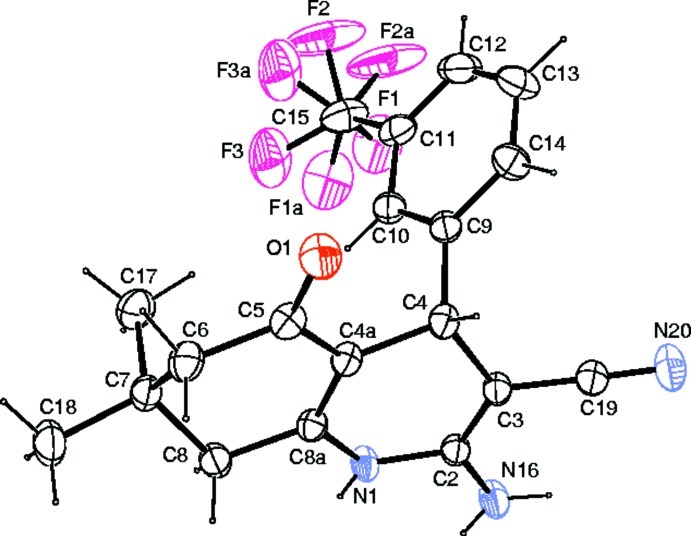
The molecular structure of the title compound with ellipsoids drawn at the 40% probability level. H atoms are shown as small spheres of arbitrary radii. The F atoms are disorded over two sets of sites.

**Fig. 2.**
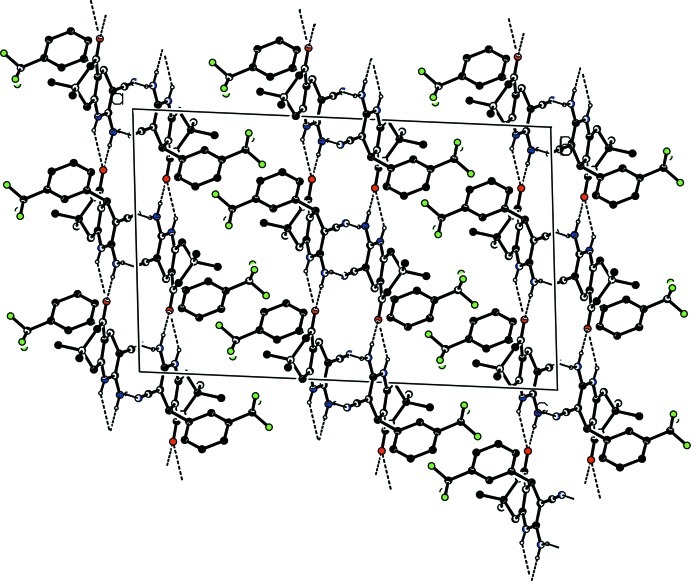
The packing arrangement of molecules viewed along the b axis. The dashed lines show intermolecular N—H···O and N—H···N hydrogen bonds. The disorder is not shown.

## supplementary crystallographic information

### Comment

The nucleus containing 1,4- dihydropyridine (DHP) nucleus act as a versatile intermediate for the synthesis of several pharmaceuticals together with those of cardiovascular drugs and as a calcium channel modulators, laser dyes and photo initiators (Leon *et al.*, 2008). The design and synthesis of 1,4-dihydropyridines has attracted much attention over the past 30 years due to the calcium antagonist effect they display (Mayler, 1989). The establishment of the pharmacological action as drugs for the treatment of cardiovascular diseases such as angina, hypertension or arrhythmia was mainly based on the structural studies carried out by X-ray diffraction on differently substituted 1,4-dihydropyridines (Triggle *et al.*, 1989). In this paper, we report the crystal structure of the title compound, (I).

In (I) (Fig.1), all bond lengths and angles are normal and correspond to those observed in related structures (Jiang *et al.*,2006; Tu *et al.*, 2005). The (N1/C2/C3/C4/C4A/C8A) and (C4A/C5—C8/C8A) rings adopt sofa conformations (ΔCs(C4) = 3.35 & ΔCs (C7) = 6.70) (Duax & Norton, 1975) with atoms C4 and C7 forming the flaps in each ring. The five essentially planar atoms (N1/C2/C3/C4A/C8A) of the dihydropyridine ring (maximum deviation 0.039 (2)Å for C8) form a dihedral angle of 88.19 (8)° with the benzene ring. The F atoms of the trifluoromethyl group are disordered over two sets of sites in a 0.840 (3):0.160 (3) ratio. In the crystal, N—H···O and N—H···N hydrogen bonds (Table 1) link molecules into a two-dimensional network parallel to (100) (Fig. 2).

### Experimental

In a 50 ml round bottom flask 5,5-dimethylcyclohexane- 1,3-dione (1 mmol) and ammonium acetate (3.5 mmol) were taken in water (10 ml). The reaction mixture was stirred at 373K for 40 min. Then malononitrile (1 mmol), 3-trifluoro methyl benzaldehyde (1 mmol) were charged, and the mixture was heated at 373K for 30 min. After the completion of reaction (monitored by TLC), the reaction mixture was stirred at RT for 15 min. The separated solid was then filtered off and recrystallized from ethanol to afford pure product as crystals suitable for X-ray diffraction. M.P.: 558–560 K, Yield: 83%.

IR(KBr): 3392, 3335, 3225, 2922, 2180, 1657, 1605, 1478 cm-1. 1H NMR (300 MHz, DMSO-d6): δ 0.87(s, 3H, CH3), 1.00(s, 3H, CH3), 2.15–2.34(dd, 2H, J= 18 Hz, CH2), 2.42–2.49(dd, 2H, J= 17.4 Hz, CH2), 4.42 (s, 1H, CH), 5.89(s, 2H, NH2), 7.38–7.55(m, 4H, Ar—H), 9.09(br s, 1H, NH).

### Refinement

All H atoms were positioned geometrically and were treated as riding on their parent C atoms, with C—H distances of 0.93–0.98 Å, N—H distances of 0.86 and with *U*_iso_(H) = 1.2*U*_eq_(C/N) or 1.5*U*_eq_(methyl C).

### Crystal data

**Table:**

C_19_H_18_F_3_N_3_O	*F*(000) = 1504
*M**_r_* = 361.36	*D*_x_ = 1.356 Mg m^−^^3^
Monoclinic, *C*2/*c*	Mo *K*α radiation, λ = 0.71073 Å
Hall symbol: -C 2yc	Cell parameters from 14640 reflections
*a* = 24.2434 (6) Å	θ = 3.4–29.1°
*b* = 9.6030 (2) Å	µ = 0.11 mm^−^^1^
*c* = 15.2426 (4) Å	*T* = 293 K
β = 93.960 (2)°	Block, white
*V* = 3540.15 (15) Å^3^	0.3 × 0.2 × 0.2 mm
*Z* = 8	

### Data collection

**Table:**

Oxford Diffraction Xcalibur Sapphire3 diffractometer	3469 independent reflections
Radiation source: fine-focus sealed tube	2462 reflections with *I* > 2σ(*I*)
Graphite monochromator	*R*_int_ = 0.059
Detector resolution: 16.1049 pixels mm^-1^	θ_max_ = 26.0°, θ_min_ = 3.4°
ω scans	*h* = −29→29
Absorption correction: multi-scan (*CrysAlis PRO*; Oxford Diffraction, 2010)	*k* = −11→11
*T*_min_ = 0.896, *T*_max_ = 1.000	*l* = −18→18
42194 measured reflections	

### Refinement

**Table:**

Refinement on *F*^2^	Primary atom site location: structure-invariant direct methods
Least-squares matrix: full	Secondary atom site location: difference Fourier map
*R*[*F*^2^ > 2σ(*F*^2^)] = 0.057	Hydrogen site location: inferred from neighbouring sites
*wR*(*F*^2^) = 0.143	H-atom parameters constrained
*S* = 1.03	*w* = 1/[σ^2^(*F*_o_^2^) + (0.0559*P*)^2^ + 3.608*P*] where *P* = (*F*_o_^2^ + 2*F*_c_^2^)/3
3469 reflections	(Δ/σ)_max_ = 0.021
247 parameters	Δρ_max_ = 0.31 e Å^−^^3^
6 restraints	Δρ_min_ = −0.39 e Å^−^^3^

### Special details

**Table:**

Experimental. *CrysAlis PRO*, Oxford Diffraction Ltd., Version 1.171.34.40 (release 27–08-2010 CrysAlis171. NET) (compiled Aug 27 2010,11:50:40) Empirical absorption correction using spherical harmonics, implemented in SCALE3 ABSPACK scaling algorithm.
Geometry. All e.s.d.'s (except the e.s.d. in the dihedral angle between two l.s. planes) are estimated using the full covariance matrix. The cell e.s.d.'s are taken into account individually in the estimation of e.s.d.'s in distances, angles and torsion angles; correlations between e.s.d.'s in cell parameters are only used when they are defined by crystal symmetry. An approximate (isotropic) treatment of cell e.s.d.'s is used for estimating e.s.d.'s involving l.s. planes.
Refinement. Refinement of *F*^2^ against ALL reflections. The weighted *R*-factor *wR* and goodness of fit *S* are based on *F*^2^, conventional *R*-factors *R* are based on *F*, with *F* set to zero for negative *F*^2^. The threshold expression of *F*^2^ > σ(*F*^2^) is used only for calculating *R*-factors(gt) *etc*. and is not relevant to the choice of reflections for refinement. *R*-factors based on *F*^2^ are statistically about twice as large as those based on *F*, and *R*- factors based on ALL data will be even larger.

### Fractional atomic coordinates and isotropic or equivalent isotropic displacement parameters (Å^2^)

**Table:**

	*x*	*y*	*z*	*U*_iso_*/*U*_eq_	Occ. (<1)
N1	0.08447 (8)	0.55967 (18)	1.03562 (11)	0.0401 (5)	
H1	0.0954	0.5379	1.0887	0.048*	
O1	0.07532 (8)	0.42797 (18)	0.73842 (10)	0.0563 (5)	
C2	0.06002 (9)	0.6875 (2)	1.01957 (13)	0.0362 (5)	
C3	0.04788 (9)	0.7283 (2)	0.93483 (13)	0.0354 (5)	
C4	0.06809 (9)	0.6491 (2)	0.85675 (13)	0.0363 (5)	
H4	0.0374	0.6442	0.8115	0.044*	
C4A	0.08252 (9)	0.5025 (2)	0.88458 (13)	0.0350 (5)	
C5	0.08524 (9)	0.3977 (2)	0.81668 (14)	0.0398 (5)	
C6	0.09892 (10)	0.2509 (2)	0.84435 (15)	0.0456 (6)	
H6A	0.1167	0.2045	0.7972	0.055*	
H6B	0.0648	0.2016	0.8531	0.055*	
C7	0.13683 (10)	0.2413 (2)	0.92868 (15)	0.0423 (5)	
C8	0.10963 (10)	0.3241 (2)	0.99962 (14)	0.0432 (6)	
H8A	0.0776	0.2732	1.0172	0.052*	
H8B	0.1355	0.3325	1.0508	0.052*	
C8A	0.09185 (9)	0.4663 (2)	0.96972 (13)	0.0356 (5)	
C9	0.11521 (10)	0.7283 (2)	0.81799 (13)	0.0396 (5)	
C10	0.16917 (10)	0.7220 (2)	0.85492 (14)	0.0430 (6)	
H10	0.1778	0.6633	0.9024	0.052*	
C11	0.21021 (11)	0.8024 (3)	0.82171 (16)	0.0512 (6)	
C12	0.19821 (14)	0.8905 (3)	0.75167 (18)	0.0651 (8)	
H12	0.2259	0.9441	0.7293	0.078*	
C13	0.14494 (15)	0.8983 (3)	0.71519 (18)	0.0704 (9)	
H13	0.1364	0.9585	0.6684	0.084*	
C14	0.10400 (12)	0.8170 (3)	0.74772 (15)	0.0547 (7)	
H14	0.0682	0.8221	0.7218	0.066*	
C15	0.26709 (13)	0.7982 (4)	0.8633 (2)	0.0745 (9)	
N16	0.04957 (9)	0.7592 (2)	1.09228 (12)	0.0525 (6)	
H16A	0.0337	0.8392	1.0877	0.063*	
H16B	0.0587	0.7251	1.1434	0.063*	
C17	0.19316 (10)	0.3023 (3)	0.91246 (19)	0.0609 (7)	
H17A	0.1884	0.3923	0.8855	0.091*	
H17B	0.2119	0.2418	0.8742	0.091*	
H17C	0.2147	0.3114	0.9674	0.091*	
C18	0.14324 (13)	0.0896 (3)	0.95821 (18)	0.0643 (8)	
H18A	0.1685	0.0845	1.0095	0.096*	
H18B	0.1573	0.0354	0.9118	0.096*	
H18C	0.1079	0.0535	0.9719	0.096*	
C19	0.01978 (9)	0.8543 (2)	0.91716 (13)	0.0392 (5)	
N20	−0.00412 (10)	0.9548 (2)	0.89909 (13)	0.0585 (6)	
F1	0.27627 (11)	0.8904 (3)	0.92719 (19)	0.1116 (10)	0.840 (3)
F2	0.30660 (11)	0.8169 (6)	0.81017 (19)	0.168 (2)	0.840 (3)
F3	0.28039 (9)	0.6777 (3)	0.9066 (2)	0.1130 (11)	0.840 (3)
F1A	0.2709 (7)	0.782 (2)	0.9463 (6)	0.1116 (10)	0.160 (3)
F2A	0.2907 (6)	0.9202 (14)	0.8416 (11)	0.168 (2)	0.160 (3)
F3A	0.2932 (6)	0.7083 (15)	0.8168 (11)	0.1130 (11)	0.160 (3)

### Atomic displacement parameters (Å^2^)

**Table:**

	*U*^11^	*U*^22^	*U*^33^	*U*^12^	*U*^13^	*U*^23^
N1	0.0578 (12)	0.0371 (10)	0.0246 (9)	0.0141 (9)	−0.0029 (8)	−0.0001 (7)
O1	0.0772 (13)	0.0577 (11)	0.0325 (9)	0.0107 (9)	−0.0065 (8)	−0.0096 (7)
C2	0.0402 (12)	0.0354 (11)	0.0329 (11)	0.0057 (9)	0.0018 (9)	−0.0005 (9)
C3	0.0388 (12)	0.0346 (11)	0.0324 (11)	0.0072 (9)	−0.0001 (9)	−0.0006 (9)
C4	0.0385 (12)	0.0411 (12)	0.0284 (10)	0.0079 (10)	−0.0046 (9)	−0.0034 (9)
C4A	0.0347 (12)	0.0378 (12)	0.0321 (11)	0.0046 (9)	−0.0020 (9)	−0.0037 (9)
C5	0.0368 (12)	0.0460 (13)	0.0362 (12)	0.0014 (10)	−0.0010 (9)	−0.0059 (10)
C6	0.0522 (14)	0.0408 (13)	0.0438 (13)	0.0045 (11)	0.0032 (11)	−0.0109 (10)
C7	0.0482 (14)	0.0377 (12)	0.0412 (12)	0.0090 (10)	0.0053 (10)	−0.0007 (10)
C8	0.0546 (14)	0.0372 (12)	0.0379 (12)	0.0058 (11)	0.0049 (10)	0.0017 (10)
C8A	0.0365 (12)	0.0364 (11)	0.0336 (11)	0.0024 (9)	0.0008 (9)	−0.0038 (9)
C9	0.0510 (14)	0.0403 (12)	0.0276 (10)	0.0084 (10)	0.0041 (10)	−0.0023 (9)
C10	0.0489 (14)	0.0468 (13)	0.0337 (11)	0.0060 (11)	0.0049 (10)	−0.0001 (10)
C11	0.0534 (16)	0.0567 (15)	0.0450 (14)	0.0006 (12)	0.0157 (12)	−0.0084 (12)
C12	0.078 (2)	0.0660 (18)	0.0545 (16)	−0.0052 (16)	0.0301 (15)	0.0029 (14)
C13	0.096 (2)	0.072 (2)	0.0451 (16)	0.0086 (18)	0.0183 (16)	0.0219 (14)
C14	0.0634 (17)	0.0645 (17)	0.0356 (13)	0.0102 (14)	−0.0001 (12)	0.0101 (12)
C15	0.0557 (19)	0.104 (3)	0.0661 (19)	−0.0103 (18)	0.0188 (15)	−0.0020 (19)
N16	0.0822 (16)	0.0441 (11)	0.0312 (10)	0.0217 (11)	0.0048 (10)	0.0007 (9)
C17	0.0431 (15)	0.0735 (18)	0.0662 (17)	0.0134 (13)	0.0044 (12)	0.0091 (15)
C18	0.093 (2)	0.0416 (14)	0.0585 (16)	0.0215 (14)	0.0101 (15)	−0.0004 (12)
C19	0.0438 (13)	0.0441 (13)	0.0289 (11)	0.0057 (11)	−0.0034 (9)	−0.0044 (9)
N20	0.0766 (16)	0.0503 (12)	0.0466 (12)	0.0247 (12)	−0.0106 (11)	−0.0038 (10)
F1	0.1020 (19)	0.117 (2)	0.111 (2)	−0.0025 (18)	−0.0302 (15)	−0.0414 (19)
F2	0.0574 (17)	0.364 (6)	0.086 (2)	−0.026 (3)	0.0359 (14)	0.015 (3)
F3	0.0607 (15)	0.105 (2)	0.169 (3)	0.0148 (13)	−0.0228 (16)	0.017 (2)
F1A	0.1020 (19)	0.117 (2)	0.111 (2)	−0.0025 (18)	−0.0302 (15)	−0.0414 (19)
F2A	0.0574 (17)	0.364 (6)	0.086 (2)	−0.026 (3)	0.0359 (14)	0.015 (3)
F3A	0.0607 (15)	0.105 (2)	0.169 (3)	0.0148 (13)	−0.0228 (16)	0.017 (2)

### Geometric parameters (Å, º)

**Table:**

N1—C8A	1.367 (3)	C10—C11	1.382 (3)
N1—C2	1.377 (3)	C10—H10	0.9300
N1—H1	0.8600	C11—C12	1.377 (4)
O1—C5	1.235 (3)	C11—C15	1.478 (4)
C2—N16	1.344 (3)	C12—C13	1.372 (4)
C2—C3	1.362 (3)	C12—H12	0.9300
C3—C19	1.406 (3)	C13—C14	1.381 (4)
C3—C4	1.522 (3)	C13—H13	0.9300
C4—C4A	1.504 (3)	C14—H14	0.9300
C4—C9	1.525 (3)	C15—F1A	1.272 (9)
C4—H4	0.9800	C15—F3A	1.308 (9)
C4A—C8A	1.348 (3)	C15—F2	1.309 (3)
C4A—C5	1.449 (3)	C15—F1	1.323 (4)
C5—C6	1.503 (3)	C15—F2A	1.355 (10)
C6—C7	1.530 (3)	C15—F3	1.360 (4)
C6—H6A	0.9700	N16—H16A	0.8600
C6—H6B	0.9700	N16—H16B	0.8600
C7—C17	1.522 (3)	C17—H17A	0.9600
C7—C8	1.528 (3)	C17—H17B	0.9600
C7—C18	1.529 (3)	C17—H17C	0.9600
C8—C8A	1.494 (3)	C18—H18A	0.9600
C8—H8A	0.9700	C18—H18B	0.9600
C8—H8B	0.9700	C18—H18C	0.9600
C9—C14	1.381 (3)	C19—N20	1.149 (3)
C9—C10	1.389 (3)		
			
C8A—N1—C2	122.02 (17)	C11—C10—C9	120.6 (2)
C8A—N1—H1	119.0	C11—C10—H10	119.7
C2—N1—H1	119.0	C9—C10—H10	119.7
N16—C2—C3	126.42 (19)	C12—C11—C10	120.5 (3)
N16—C2—N1	114.42 (18)	C12—C11—C15	119.3 (3)
C3—C2—N1	119.14 (18)	C10—C11—C15	120.1 (2)
C2—C3—C19	119.98 (19)	C13—C12—C11	119.4 (3)
C2—C3—C4	122.51 (18)	C13—C12—H12	120.3
C19—C3—C4	117.28 (18)	C11—C12—H12	120.3
C4A—C4—C3	109.16 (17)	C12—C13—C14	120.2 (3)
C4A—C4—C9	114.22 (18)	C12—C13—H13	119.9
C3—C4—C9	110.12 (17)	C14—C13—H13	119.9
C4A—C4—H4	107.7	C9—C14—C13	121.3 (3)
C3—C4—H4	107.7	C9—C14—H14	119.4
C9—C4—H4	107.7	C13—C14—H14	119.4
C8A—C4A—C5	119.74 (19)	F1A—C15—F3A	116.9 (12)
C8A—C4A—C4	122.22 (18)	F1A—C15—F2	128.8 (8)
C5—C4A—C4	118.03 (18)	F2—C15—F1	105.7 (4)
O1—C5—C4A	120.7 (2)	F1A—C15—F2A	110.3 (11)
O1—C5—C6	121.1 (2)	F3A—C15—F2A	102.1 (10)
C4A—C5—C6	118.14 (19)	F2—C15—F3	104.9 (4)
C5—C6—C7	113.60 (18)	F1—C15—F3	101.0 (3)
C5—C6—H6A	108.8	F1A—C15—C11	115.6 (8)
C7—C6—H6A	108.8	F3A—C15—C11	104.8 (7)
C5—C6—H6B	108.8	F2—C15—C11	115.6 (3)
C7—C6—H6B	108.8	F1—C15—C11	113.7 (3)
H6A—C6—H6B	107.7	F2A—C15—C11	105.7 (8)
C17—C7—C8	110.5 (2)	F3—C15—C11	114.5 (3)
C17—C7—C18	109.9 (2)	C2—N16—H16A	120.0
C8—C7—C18	109.12 (19)	C2—N16—H16B	120.0
C17—C7—C6	109.5 (2)	H16A—N16—H16B	120.0
C8—C7—C6	107.43 (18)	C7—C17—H17A	109.5
C18—C7—C6	110.3 (2)	C7—C17—H17B	109.5
C8A—C8—C7	112.92 (18)	H17A—C17—H17B	109.5
C8A—C8—H8A	109.0	C7—C17—H17C	109.5
C7—C8—H8A	109.0	H17A—C17—H17C	109.5
C8A—C8—H8B	109.0	H17B—C17—H17C	109.5
C7—C8—H8B	109.0	C7—C18—H18A	109.5
H8A—C8—H8B	107.8	C7—C18—H18B	109.5
C4A—C8A—N1	121.11 (19)	H18A—C18—H18B	109.5
C4A—C8A—C8	123.73 (19)	C7—C18—H18C	109.5
N1—C8A—C8	115.15 (18)	H18A—C18—H18C	109.5
C14—C9—C10	118.1 (2)	H18B—C18—H18C	109.5
C14—C9—C4	119.7 (2)	N20—C19—C3	176.9 (2)
C10—C9—C4	122.06 (19)		
			
C8A—N1—C2—N16	172.0 (2)	C2—N1—C8A—C4A	9.3 (3)
C8A—N1—C2—C3	−6.8 (3)	C2—N1—C8A—C8	−169.6 (2)
N16—C2—C3—C19	−2.7 (4)	C7—C8—C8A—C4A	21.7 (3)
N1—C2—C3—C19	176.0 (2)	C7—C8—C8A—N1	−159.39 (19)
N16—C2—C3—C4	171.7 (2)	C4A—C4—C9—C14	141.8 (2)
N1—C2—C3—C4	−9.7 (3)	C3—C4—C9—C14	−95.0 (2)
C2—C3—C4—C4A	21.0 (3)	C4A—C4—C9—C10	−42.8 (3)
C19—C3—C4—C4A	−164.59 (19)	C3—C4—C9—C10	80.4 (2)
C2—C3—C4—C9	−105.2 (2)	C14—C9—C10—C11	−0.2 (3)
C19—C3—C4—C9	69.3 (2)	C4—C9—C10—C11	−175.6 (2)
C3—C4—C4A—C8A	−18.5 (3)	C9—C10—C11—C12	0.3 (4)
C9—C4—C4A—C8A	105.2 (2)	C9—C10—C11—C15	178.0 (2)
C3—C4—C4A—C5	160.28 (18)	C10—C11—C12—C13	0.2 (4)
C9—C4—C4A—C5	−76.0 (2)	C15—C11—C12—C13	−177.5 (3)
C8A—C4A—C5—O1	178.0 (2)	C11—C12—C13—C14	−0.9 (4)
C4—C4A—C5—O1	−0.9 (3)	C10—C9—C14—C13	−0.5 (4)
C8A—C4A—C5—C6	−0.4 (3)	C4—C9—C14—C13	175.1 (2)
C4—C4A—C5—C6	−179.2 (2)	C12—C13—C14—C9	1.1 (4)
O1—C5—C6—C7	150.7 (2)	C12—C11—C15—F1A	143.6 (11)
C4A—C5—C6—C7	−31.0 (3)	C10—C11—C15—F1A	−34.2 (11)
C5—C6—C7—C17	−65.5 (3)	C12—C11—C15—F3A	−86.1 (9)
C5—C6—C7—C8	54.5 (3)	C10—C11—C15—F3A	96.1 (9)
C5—C6—C7—C18	173.4 (2)	C12—C11—C15—F2	−34.1 (5)
C17—C7—C8—C8A	70.0 (3)	C10—C11—C15—F2	148.1 (4)
C18—C7—C8—C8A	−169.0 (2)	C12—C11—C15—F1	88.4 (4)
C6—C7—C8—C8A	−49.4 (3)	C10—C11—C15—F1	−89.3 (4)
C5—C4A—C8A—N1	−173.7 (2)	C12—C11—C15—F2A	21.3 (8)
C4—C4A—C8A—N1	5.1 (3)	C10—C11—C15—F2A	−156.5 (8)
C5—C4A—C8A—C8	5.1 (3)	C12—C11—C15—F3	−156.2 (3)
C4—C4A—C8A—C8	−176.1 (2)	C10—C11—C15—F3	26.0 (4)

### Hydrogen-bond geometry (Å, º)

**Table:**

*D*—H···*A*	*D*—H	H···*A*	*D*···*A*	*D*—H···*A*
N1—H1···O1^i^	0.86	2.39	3.117 (2)	143
N16—H16*A*···N20^ii^	0.86	2.12	2.966 (3)	168
N16—H16*B*···O1^i^	0.86	2.08	2.897 (2)	158

Symmetry codes: (i) *x*, −*y*+1, *z*+1/2; (ii) −*x*, −*y*+2, −*z*+2.
